# STRENGTH IN AGE: INCREASING THE POWER OF FAMILY CAREGIVERS

**DOI:** 10.1093/geroni/igz038.1644

**Published:** 2019-11-08

**Authors:** Heather Young, Theresa Harvath, Fawn Cothran

**Affiliations:** Betty Irene Moore School of Nursing - UC Davis, Sacramento, California, United States

## Abstract

This session addresses approaches to strengthening the capacity of family caregivers in the context of intense and complex care. Recognizing the increasing role that families play in delivering complex care at home to individuals with multiple conditions, this symposium highlights approaches to enhancing support and increasing the power of family caregivers. In the first half of the symposium, the papers elucidate characteristics of the caregiving situation that put caregivers at risk and suggest potential areas for intervention by health systems. The second half explores system level approaches to enhancing capacity for family caregivers. On the demand side, the first paper will examine the social network of family caregivers, highlighting effects of social isolation on caregiver health. The second paper uses national data to understand the relationship between higher demand caregiving situations and the strain and challenges that caregivers experience. On the potential solutions side, the third paper addresses a collaborative design of an intervention to enhance supports for family caregivers of persons with dementia at a critical time, during hospitalization. The last paper provides an overview of evidence-based technological solutions to support family caregiving. Taken together, these papers establish some of the demand characteristics of the caregiving situation and provide potential health system solutions to improve capacity of family caregivers. In the final segment, three discussants will reflect on the implications of these papers for clinical practice, education, research and policy.

